# Editorial: Reading, literature, and psychology in action

**DOI:** 10.3389/fpsyg.2022.1068030

**Published:** 2023-03-08

**Authors:** Philip Davis, Rhiannon Corcoran, Josie Billington, Arthur Frank

**Affiliations:** ^1^Centre for Research into Reading, Literature and Society, University of Liverpool, Liverpool, United Kingdom; ^2^Department of Primary Care and Mental Health, School of Psychology, University of Liverpool, Liverpool, United Kingdom; ^3^English Department, School of the Arts, University of Liverpool, Liverpool, United Kingdom; ^4^Department of Sociology, University of Calgary, Calgary, AB, Canada

**Keywords:** literature, psychology, reading therapy, mental health, bibliotherapy

## Introduction

In asserting that the reading of literature is psychology in action we mean many things: from seeing individuals and groups work through complex social scenarios as they consider and contemplate feelings, memories and problems, and weigh up options and alternatives within the comfort of their own minds; to watching the benefit that mastering “hard” texts brings to those who previously believed they could not do so. The gaining of what we might easily call “confidence,” the movement from passivity to mental action, has the power to catalyze change beyond the literary, into the taking of life opportunities. Thus, we propose that the practice of shared reading in groups where there is opportunity to learn about other minds, to assess and contrast your thoughts and feelings to those of others provides a live and explicit demonstration of minds interacting amid a shared mission and with a combined purpose. A real showcasing of the skill that psychologists call “Theory of Mind,” first introduced by Premack and Woodruff (1978). In shared reading settings there is both the opportunity to represent several other's cognitive and emotional states and to judge your responses against those of others with a view to appraising or re-appraising them. With several authors now interested in the role that reading fiction can play in honing socio-cognitive and empathic skills (e.g., Mar et al., 2006; Kidd and Castano, 2013), the door to the use of fiction as an option for those whose theory of mind skills are argued to be compromised is open.

In an interview conducted in 2016 for the Washington Post, Keith Oatley described books as “life simulators” (Kaplan, 2016). For us, this term reaches into the heart of what literature can do. By providing the opportunity to experience the world with others in simulatory or “as if” mode we can consider life's experiences from a less directly problem-oriented, more metaphorical stance. In so doing, we engage a less well-used type of information processing, perhaps a more creative form of thinking that triggers more than common or garden problem solving, allowing freer rein to consider looser, novel, more dispersed connections and possibilities (Bottini et al., 1994; Diaz and Eppes, 2018).

Further, the nature of literary fiction simulates a life that many readers may never themselves have the opportunity to experience directly. Other worlds are uncovered with new thoughts and feelings released by them, encouraging mental flexibility and a more open emotional consideration of life events, yours and others, generally. This has been one finding of the research conducted by the Center for Reading Literature and Society, a collaboration of English literature specialists, psychologists, and medical academics and practitioners at the University of Liverpool, researching the effects on the human psyche of reading serious literature. In the Centre's studies with its practice partner, The Reader, which delivers over 600 reading groups in community, clinical and secure settings in the UK and beyond, this release of new thoughts and the broadening of emotional responses to life's experiences as well the invaluable opportunity to reason analogously (in “as if” mode) have been witnessed time and again in action during reading groups involving people with common mental health, substance use issues, chronic pain and dementia for example (https://www.thereader.org.uk/about-us/our-research/).

But for now, let us more fully illustrate the power of literary reading amid the context of adversity and trouble. Adverse Childhood Experiences (ACEs) are much more common than we like to acknowledge and their toll on wellbeing, mental and physical health underpins some of the enduring inequities of health, life expectancy and healthy life expectancy (Bellis et al., 2013). This is because, being rooted in childhood, they are experiences set, like landmarks and follies, within our information processing system, governing how we see the world and how we navigate our way through it across the life course. As intensely emotional experiences, they have the power to bias memory and executive functioning systems (Ibrahim et al., 2022) and, in so doing, set a course to life that aims to protect the self by establishing a way of tackling the world to avoid further adversities.

In this context of ACES and their cognitive and emotional legacy, we employ “safety behaviors,” as psychologists call them—avoidant strategies that serve us well in the short term by side-stepping further trauma. While these behaviors and strategies work well to keep us out of harm's way in the short term, they generally steer us poorly into and through adult life (McManus et al., 2008). The default decision-making style is one that is founded on the anticipation of unhappy outcomes and our behaviors, guided by this style, ramp up the manner and extent to which we keep ourselves safe, at least for the time being. This channeling of permitted experience almost inevitably leads to isolation and to lives less than well-lived—a predetermined route whose course is set by initial adversity and the automatic prediction of further danger. Things become set and, as a result, we become stuck in a particular way of understanding and dealing with the world.

In illustrating the power of literature to mitigate adversity, specific case-histories provide the most vital initial evidence. Someone, who we'll call Hattie, described her experience of ACES and how reading helped her in a piece of writing sent to one of us:

*In my room again, trying not to listen too carefully to their argument. Angry, loud words—“immature,” “leaving,” “the children” and then, the slamming door—work as arrows to pierce my flimsy shield. These words do break my bones. But I manage to stay alive, scathed but still here…to overhear another bout. Maybe later today… or tomorrow. Most likely though I'll be tip-toeing amongst the tense silent wreckage of this discorded house, resting there for some unknowable time to come. She goes to her bed, he to his bottle*.

Happily, Hattie's adult life has been, on the face of it at least, a successful one. Something protected her beyond her own safety behaviors. Here, she tells us what that is:

*It was these times when I read. We had loads of faded orange and white Penguin classics on bookshelves here and there about the house. Both parents read when they weren't themselves starring in “Who's Afraid of Virginia Woolf?”*.

*I was lucky that way—not just finding mental escape but making some alternative sense of things within the house from which I was escaping. I was too scared to escape by actually leaving—in case things got better, or in case I missed even the yellings which I thought were somehow my fault and my responsibility*.

*Thomas Hardy's landscapes played their part in the rescue of me, and D.H. Lawrence provided my nourishment. The finding that words could be used like that; not just to break bones but to soothe and to open-up. Making me ready to be vulnerable… in another place. Coming to understand that lives were supposed to be hard, amongst the love and the land. Drifting away to join Hardy's Tess and Lawrence's Gypsy. These folk filled a void where familial comfort should have been*.

But at university Hattie studied psychology rather than literature, for being more ostensibly relevant to the situation in which she had grown up. Indeed she later became a psychologist. She was like many young people pulled into the study of psychology because of the things that have happened to them. But she now says, “*While psychology promises that, it too often fails to deliver.”*

From her own traumatic background, Jeanette Winterson studied literature at Oxford, a reader who then became a writer. In her autobiography, *Why Be Happy When You Could Be Normal?* (Winterson, 2011) she advocates the power of fiction rather than fact, though her adopted mother had banned novels because those were the dangerous books “there was trouble in” (p. 37):

*Reading things that are relevant to the facts of your life is of limited value. The facts are, after all, only the facts, and the yearning passionate part of you will not be met there. That is why reading ourselves as a fiction as well as fact is so liberating. The wider we read the freer we become. (p. 117)*

*I had no one to help me, but the T. S. Eliot helped me*.

*So when people say that poetry is a luxury, or an option, or for the educated middle classes, or that it shouldn't be read at school because it is irrelevant, or any of the strange stupid things that are said about poetry and its place in our lives, I suspect that the people doing the saying have had things pretty easy. A tough life needs a tough language—and that is what poetry is. That is what literature offers—a language powerful enough to say how it is. It isn't a hiding place. It is a finding place. (p. 40)*

Poetry wasn't merely a soft flowery language offering escapism: the language was as powerful—in triggering emotions and memories, and in stimulating responsive mental faculties—as the obstacles and pains the young woman suffered. “Turn your fear into a safeguard,” an equivalent young woman is urged in George Eliot's *Daniel Deronda* (Eliot, 1876/1998): “We are not always in a state of strong emotion, and when we are calm we can use our memories and gradually change the bias of our fear, as we do our tastes. Take your fear as a safeguard. It is like quickness of hearing. It may make consequences passionately present to you. Try to take hold of your sensibility, and use it as if it were a faculty, like vision” (chapter 36).

If the reading of literature has the power to alter minds and change lives in the way Hattie the psychologist and Jeanette the novelist describe, then how it does so becomes a question in which the discipline of psychology ought to be interested. However, sensitively to address and develop this question, the standardized understandings of psychology need to mingle with a deep knowledge of literature and the practice of reading. Transdisciplinary scholarship and practice can produce better informed, grounded models and theories that improve our understanding of humanity at its best even while struggling amidst its worst.

Within their own realms, we allege that both Psychology and English literature have become closed in and rigidified by their own brands, methods and approaches. Both need opening up to enjoy the fresh air of cross-talk. Graduates of both disciplines deserve the chance to see reading and psychology in action. In both, as also in students of philosophy, the will to make sense of things (Dennett, 1998) is or should be the prompt and center of study. In advocating an academic seesaw, we see the interaction between literature and psychology as a site for practice informing, challenging and revising theory as much as theory informing, challenging and revising practice.

This Research Topic concentrates on the benefits to psychology, both as a formal discipline (with a capital P) and as the arena of all human need. Here we claim that the interaction between psychology and literature demonstrated in these pages is for the benefit of Psychology itself by the challenge of its often simplified orthodoxies, its insufficiently tested pillars, its imprisoning boxes and arrows—and the temptation of its too ready acceptance of the efficient information-processing mission. In their papers, both Chapple et al.^1^ and Andersen, in relation to autistic people and people suffering from cancer, respectively, challenge the model of “efficiency”: that idea of information-processing and data-reduction, in the economy of summary and paraphrase and labeling, which must once have offered human beings an advantage in their evolution. But now the defaults and the literalisms threaten our understanding of complexity, our emotional intelligence and a true educational development. Through literature however, as an unsettling model of felt human experience in action, we can learn or relearn the full complexity of individual differences; reversing the trend to diminish experience into spectacularly un-interesting, but easily explainable, dogma. In part, we can think of this as un-sciencing psychology to permit again an engagement with its full richness.

The research of the Center for Reading, Literature and Society—often conducted, as Harsh explains, in knowledge exchange with outreach program The Reader (https://www.thereader.org.uk)—has been pursuing these transdisciplinary working practices for over a dozen years. Some of the papers collected in this Research Topic reflect this work, while others come from colleagues further afield whose interests are aligned. Collectively these authors pursue answers to what the reading of literature does, can do, and how it does it. It has a particular focus on the debunking of rigid, normative ideas. Besides Winterson's own target (that poetry being arty and elitist is only a pastime “*for the educated middle classes*”), these include other assumptions and biases:

- That mental distress involves wrong thinking coming from a somehow inherently compromised brain;
- That poor understanding and expression of emotion, and restricted feeling for and with others are emblematic of some “neurodevelopmental conditions” or “personality disorders”;
- That the engagement of the emotions is automatically irrational, meaning that a top-down approach to life is obviously to be preferred over a bottom-up one, even as we know that information-processing has to be a balanced flow of both.

In this issue, Devereux explores the uses of uncertainty in the interaction between her study of neuropsychology and the writing of her own fiction. Green considers the relation of reading literature to the experience of loss and grief. Chapple et al. (see text footnote 1) investigates emotional and imaginative intelligence amongst autistic readers customarily stereotyped and stigmatized as lacking in empathy. The papers examine reading and the human situation across a wide range of contexts:

- In different settings, with Tangeras in a care home for adults living with dementia, Watkins et al. in a high secure hospital, and Andersen among cancer patients onsite and on-line,
- At different ages, starting from the work on childhood reading in Kuzmičová et al. in Czechia, and Zheng et al. in a Chinese rural setting; through to diverse community reading groups of different ages; and to care homes for the aged,
- Through varied methods, including Kuzmičová et al. on Q methodology, Davis et al. on eye-tracking, and Tschense and Wallot using Confirmatory Factor Analysis, and a range of literary and linguistic analyses,
- Across different disciplines, with Whistler on the relation of reading to thinking in the field of philosophy, and Harsh on belief, religion and theology with particular relation to the shared reading of the novels of Marilynne Robinson.

But what, firstly and finally, we wish to establish in this introduction is a sense of the “liveness” of reading, as a dynamic testing-ground for thinking about human existence *in situ*, without knowing-in-advance, and thus what we mean by this constituting psychology in action.

## The safe space of literature

When human vulnerability meets a cherished character or vital situation in a book, it discovers a safety to explore without the threat of “real” life, though as close as possible to it. Fiction including poetry allows us to explore the “what might happen.” In literature we have the safe space to skilfully hold and work through, by reading, the several possibilities and their likely implications in parallel (Snell and Grainger, 2019). You can regard, consider and come to value the uncertainties of life, secure on the other side of the page, while walking, hand-in-hand with real humanity (Carleton et al., 2012). Literature becomes a model of real life where we can find fully formed friends and enemies and anomalous individuals of whom we can ask questions, and to whom put questions. We meet others who deal with things in so many different ways and we get to see the consequences of their actions. The not knowing what will happen, how someone will react, what may come in sideways to disrupt a situation: these are all real. This is what happens in life. Good literature packs a whole lifetime of feeling, of experience, of uncertainties into a few undisturbed hours: hence the necessary power, invention and mobility of its responsive language, as distinct from a static language of names and labels and boxes; hence the literary compression's explosive effect on readers. This is not the same as the dramas and melodramas of much television that lay things out in order to reach and often condescend to the many (as in mass media). Instead, it is the discovery, at times the rescuing, of the self by the self, mentored by a great figure who is not a teacher, nor a parent or grandparent. Neither a boss nor a therapist. Nor a psychologist. Instead, an author, who does not tell us what to think, nor even how to think; but through the novel, short story or poem, presents episodes and situations for us individually to consider, triggering our own autobiographical experience, implicitly or explicitly, as a vital part of the immersed, contemplative experience of reading.

## A starting place for rich learning

Developmental psychologists tell us that we learn best from those we consider “like me” (Perner et al., 1994). So, perhaps literature does its best work initially when we recognize a little of ourselves in the literary figure and can “get into” the work that way. This is a starting place, where being involuntarily touched and moved by the circumstances and reactions of another whom we see as “like us” in some if not most ways, can trigger the opening up of alternative or reclaimed ways of doing, seeing and understanding beyond that which is already established, engrained, habitualized or repressed. In this place, we feel first and then we reflect. By contrast day-to-day life experiences can become plodding, too slow, too anticipated, too finished before they have started, too convenient. We react within it in a safe way that minimizes the inner resources we have to allocate to it, discouraging too much emotional challenge, over-protective of resources and security. In experiencing life like this we rest on determined agendas, defaults, ruminative generalizations and opinions set in advance. “Convenience! Convenience!” writes D. H. Lawrence indignantly in an essay “The Novel and the Feelings” (Lawrence, 1925/1988): “There are convenient emotions and inconvenient ones. The inconvenient ones we chain up, or put a ring through their nose. The convenient ones are our pets.” The reading of literature involves far more feeling and thought of what is untamed, inconvenient and dynamic.

## Keeping novelty

There are occasions in life, such as bereavement or falling in love that act upon us as literature does. In fact, during these circumstances, we can feel we are a character in an uncompleted and not fully legible book—almost watching and reading our own lives, or trying to do so. These are the rare, seldom-had events for which we have no ready script and so, for which, our higher order processing brain is ill-equipped (Norman and Shallice, 1986). Here feeling and emotion must step in to guide us through the disrupted, un-navigated landscape that we find ourselves amid. Amongst these are the glory days, the days we don't forget; the days our memory does its work on, cataloging and networking within our information-processing system for later use. With a script, psychologists say, we can guide ourselves more rationally, directly and efficiently though the next time. But as we reduce down into efficient processing systems, we lose the beauty of the un-understandable, the un-knowable, the feelings we have no ready words to describe, the embodied experiences, the topsy-turviness of where our mixed emotions throw us. In its special places, when things land on us unexpectedly, literature gives this back to us but in a kind and leveling way. In a way that ensures we don't become over-whelmed—just “whelmed.” The literary script—no programme but always a fresh endeavor—is a repository through which we can experience or re-think life's rare, neglected or repressed events and our reactions to them.

## A community of reading

If we believe, as we do, that reading literature has an important relation to mental health, it is no good simply prescribing books to people who for various reasons will probably not read them. In the effort to attract and reach people who would not otherwise read novels or poetry, imagine, not reading literature alone, but reading literature live together in a group, conducted with the help of an experienced literary reader. How many formerly separate lives align across and within the novel just as within the group? How many similarities, differences, affordances, and developments become possible as our physiology and our mental responsiveness align with those who, in the process, may become “our” others? Community is created at deep personal levels when literature is read “live” together, creating a “we-ness” in and through literature (Gallotti and Frith, 2013). In this setting the novel or poem becomes the focus, the point and voice of human similarity, the shared interest from which other felt interests can organically spring. In live group reading we peer not only into characters' lives, but also into those of our fellow readers. These folk can become fellow-encouragers, valuing our attempts to make sense by acknowledging feelings and memories, and finding a way of talking and thinking about them. Live reading groups can build a eudaimonia that is often only now experienced in life through meaningful work and career, not enjoyed by many.

In this issue, we draw attention to matters that are not just one discipline's concerns and to a practice that is not just one carry-out idea. Education through praxis is the sole thing that will enable people to get used to this way of thinking and being, where being a reader is to be a seeker for meaning. As if instead of suffering passively we can use what we have had and what we have not had, to be the novelists or poets or therapists of ourselves, as Winterson put it.

## Author contributions

PD and RC wrote the first draft of the manuscript. JB and AF read and commented upon the manuscript. All authors have read and approved the final version.

## Footnotes

^1^Chapple, M., Davis, P., Billington, J., and Corcoran, R. (under review). Exploring the different cognitive, emotional and imaginative experiences of autistic and non-autistic adult readers when contemplating serious literature as compared to non-fiction.
